# Is there a comparable Mp-MRI for incidental prostate uptake on 18 F-FDG PET/CT?

**DOI:** 10.1186/s12957-024-03578-0

**Published:** 2024-12-20

**Authors:** Merve Şam Özdemir, Nurullah Kaya, Metin Savun, Emin Taha Keskin, Sabahattin Yüzkan, Fatma Zeynep Arslan, Burcu Budak, Özgür Omak, Aytül Hande Yardımcı, Harun Özdemir

**Affiliations:** 1 Department of Radiology, Başaksehir Çam and Sakura City Hospital, Istanbul, Turkey; 2 Department of Urology, Başaksehir Çam and Sakura City Hospital, Istanbul, Turkey; 3 Department of Radiology, Koc University, Istanbul, Turkey; 4 Department of Nucleer Medicine, Başaksehir Çam and Sakura City Hospital, Istanbul, Turkey

**Keywords:** Prostate cancer, 18F-FDG PET-CT, Multi-parametric MRI, İncidental Prostate 18F-FDG Uptake

## Abstract

**Purpose:**

Although 18 F-FDG-PET/CT is helpful in defining many types of cancer, localized prostate cancer should not be treated with this technique. This study describes the use of multi-parametric MRI (mpMRI) to characterize incidental 18 F-FDG uptake in the prostate.

**Methods and Materials:**

While 18 F-FDG-PET/CT is useful for characterizing a variety of cancers, it is not advised for prostate cancer that is localized. This work investigates the use of mpMRI to describe incidental 18 F-FDG uptake in the prostate.mpMRI included T2-weighted (T2W), dynamic contrast enhancement (DCE), and apparent diffusion coefficient (ADC) sequences. Patients were classified according to PI-RADS (Prostate Imaging Reporting and Data System) version 2.1 by an experienced uroradiologist, and 18 F-FDG-PET was evaluated to determine whether the area of involvement on CT had a counterpart in mpMRI. A biopsy was performed on 30 of the 92 patients. These patients’ maximum standardized uptake values (SUVmax) 6 < and ≥ 6, PS(PSA) density 0.15 < and ≥ 0.15, PSA level, uptake pattern (focal involvement/diffuse involvement), and PI-RADS scores were compared. *P* < .05 was considered statistically significant. Logistic regression was used to analyze PI-RADS score groups age, PSA, PSA density and SUVmax.

**Results:**

In the study, 92 patients with incidental 18 F-FDG-PET/CT prostate uptake were examined. Median age was 66, PSA median was 3.6 ng/ml (range: 0-3198 ng/ml). Notably, in 70.6% of cases, PET/CT uptake didn’t correlate with mp-MRI findings. Among PI-RADS 3-4-5 patients (29.3%), there was a correlation. Biopsies in 30 patients revealed 43.3% benign, 56.7% malignant. Significant differences between benign and malignant cases were observed in PSA density, PI-RADS scores, and PSA levels (*p* < .05), while SUVmax and uptake pattern were not significant. In multivariate logistic regression analysis, PI-RADS score groups were found to be independent risk factors for predicting malignancy.

**Conclusions:**

Our study showed that incidental 18 F-FDG-PET/CT prostate uptake was detected and that high PSA density values, PI-RADS scores, and PSA values, such as in routine patients, and not PET-CT findings such as SUVmax and uptake pattern, were more predictive of malignancy.

**Supplementary Information:**

The online version contains supplementary material available at 10.1186/s12957-024-03578-0.

## Background

Various types of cancer are routinely staged using 18 F-fluorodeoxyglucose positron emission tomography-computed tomography (18 F-FDG PET-CT) because metabolically active tumours typically use the glycolytic pathway [1]. There is disagreement in the current guidelines staging the use of 18 F-FDG PET-CT in staging prostate cancer (PCa) despite its frequent use in staging various types of cancer [2]. Since 18 F-FDG PET-CT is not typically recommended as an imaging method for male patients with suspected prostate cancer, there is sparse radiological data on its use in the early staging of primary prostate cancer [2]. Additionally, 18 F-FDG can complicate the evaluation of the prostate gland due to the spillover of activity from the adjacent bladder and potential residual urine in the prostatic urethra. Jadvar et al. stated that 18 F-FDG PET-CT can serve as a biomarker in castrate-resistant metastatic PCa; however, its use in primary disease remains uncertain [3]. The use of 18 F-FDG-PET-CT has increased over the years. It is commonly used for the initial evaluation of metastases of unknown primary origin, treatment planning and monitoring therapeutic response. Additionally, it is frequently employed in assessing high-risk cases for malignancy and evaluating disease recurrence. Given these indications, incidental focal uptake in the prostatic gland can be encountered in radiology practice during an 18 F-FDG PET-CT scan. Characterising these increased focal 18 F-FDG uptake areas will contribute to effective diagnosis and treatment processes. Given that prostate cancer is the most common type of cancer in male patients, a detailed analysis of these incidental areas will significantly impact treatment decision-making processes [1].

Serum prostate-specific antigen (PSA) levels and digital rectal examination (DRE) are the most widely used diagnostic techniques for identifying prostate cancer. However, both approaches have less than ideal accuracy. PSA’s specificity is extremely low, as prostate cancer is not present in 70–80% of people with PSA levels over the standard clinical threshold level (4 ng/mL) [4, 5]. Prostate MRI applications have come a long way in the last 20 years, and PI-RADS(Prostate Imaging Reporting and Data System) based on multiparametric magnetic resonance imaging (mpMRI) is now often employed in clinical practice. The pooled sensitivity and specificity for PI-RADS v2.1 were 0.87 and 0.74, respectively, according to a meta-analysis of 10 studies [6].

During our radiology practice, we incidentally observed areas of increased 18 F-FDG uptake in the prostate gland when 18 F-FDG PET-CT was used for any other any cancer staging or investigation. This study investigates the effectiveness of mpMRI in non-invasively characterising and determining the potential clinical significance of these incidentally identified lesions. To the best of our knowledge, our study is the first to measure the MRI equivalent of incidental prostate involvement detected on an FDG taken for any reason and the value of predicting the presence of cancer histopathologically with the PI-RADS score. Additionally, it aims to analyse the correlation between PSA density, SUVmax (maximum standardised uptake values) and histological data.

## Methods

### Study design and patient population

This single-centre retrospective study was approved by the Basaksehir Cam and Sakura City Hospital local institutional review board (E96317027-27/12/2023). The eligibility of male patients who underwent both prostate mpMRI and 18 F-FDG PET-CT examinations for any reason (cancer investigation or staging) between March 2020 and March 2024 was determined. Patients who had previously received treatment for prostate cancer and who had incomplete or poor-quality mpMRI scans were not included in the study. A total of 5,305 prostate mpMRIs were retrospectively scanned, and those with PET-CT results and those with biopsy results were separated.

A total of 92 patients who had undergone scans using both imaging methods were identified. Thirty of these patients had histopathological diagnoses of prostate lesions obtained from either a cognitive transrectal ultrasound (TRUS) fusion-guided biopsy or a transperineal fusion biopsy.

### Image acquisition and processing

All MRI examinations were performed on a 3.0 Tesla MRI scanner (Verio, Siemens, Erlangen, Germany) using sequences according to the PI-RADS v2.1 protocol [7]. For contrast-enhanced T1-weighted imaging (T1ce), a bolus of 0.1 ml/kg of Gadobutrol (Gd-BT-DO3A, Gadovist^®^, Bayer Schering Pharma AG, Berlin, Germany) was intravenously administered. Whole-body 18 F-FDG PET-CT imaging was performed using a fully integrated PET and CT scanner (Philips Ingenuity TF PET-CT). Prior to imaging, all patients fasted for at least 4 h. Serum glucose levels measured during 18 F-FDG injections were below 200 mg/dL for all patients. Approximately 60 min later, a low-dose CT scan was performed using the following parameters: 110–160 mAs, 120–140 kV and slice thickness of 4 mm. Subsequently, a positron emission scan was acquired in the same transverse field of view with a bed position of 2.5 min per position in the craniocaudal direction. The total scan duration per patient was approximately 15 min. CT images were used for attenuation correction, and all images were reconstructed and stored in axial, coronal and sagittal sections.

Image processing was performed at a dedicated workstation (Philips Intellispace Potal-Mirada). A semi-automatic ellipsoid-shaped volume of interest (VOI) was drawn around the primary prostatic lesion, including the entire lesion in the axial, sagittal and coronal planes. Automatically generated by the software on the workstation, SUVmax value were recorded.

### Image analysis

An experienced nuclear medicine physician (Ö.O), blinded to histology and clinical information, retrospectively identified suspicious areas in attenuation-corrected 18 F-FDG PET-CT scans. VOIs were manually drawn to identify areas where SUVmax could be calculated. SUVmax values were recorded for each area of increased 18 F-FDG uptake in the prostate, as well as for the entire prostate, for each patient. The mpMRI data were evaluated for target lesions as documented in each patient’s prostate MRI report. PI-RADS scores were independently assessed by a radiologist with at least 5 years of experience in mpMRI reporting (more than 5,000 mpMRI reports) (M.Ş.Ö). According to PI-RADS v2.1, PI-RADS scores, PSA values and PSA density were stated in the reports.

The lesions that were positive on 18 F-FDG PET-CT were evaluated for the presence or absence of lesions identified by the radiologist in the same localisation on mpMRI. Based on suspicious lesions identified on 18 F-FDG PET-CT scan images and mpMRI cognitive fusion or transperineal fusion biopsy was performed on 30 patients.

### Statistical analysis

The Statistical Package for the Social Sciences version 24 (SPSS IBM Corp.; Armonk, NY, USA) program was used for the data analysis. Independent t-tests, chi-square tests, and Fisher’s exact tests were used to compare the groups. Quantitative data are expressed as median. Categorical data were expressed as n (frequency) and percentages (%). Data were analyzed at a 95% confidence level, and *p* value was considered significant if less than 0.05. Multivariate logistic regression (LR) analysis was used to evaluate the accuracy of the parameters.

## Results

As a result, 92 patients with areas of increased 18 F-FDG uptake in the prostate gland were included. The median age of the patients was 66 (range: 23–90), and the median PSA value was 3.6 (range: 0–3,198) (Table 1). In 65 patients (70.7%), there was no radiological counterpart on mpMRI for the area with the incidental FDG uptake increase identified using 18 F-FDG PET-CT. The area of involvement corresponded to the central zone in 4 patients and to benign areas such as the transurethral resection defect in one patient. Only 27 patients (29.3%) had a radiological counterpart in mpMRI for the lesion identified using 18 F-FDG PET-CT, noted as PI-RADS 3, 4 or 5. PSA, PSA density and PI-RADS scores are described in detail in Table 2. Biopsy results of 30 patients who underwent biopsy, 13 (43.3%) were benign and 17 (56.7%) were malignant. 17 patients with malignant biopsy results, 16 (94%) had acinar adenocarcinoma and 1 (6%) had ductal adenocarcinoma (DAC). Pathological diagnosis was reported as granulomatous prostatitis for two of the benign lesions, and these lesions had high PI-RADS scores, PSA, PSA densities and SUVmax (≥ 6) values. When comparing patients whose biopsy results were reported as malignant or benign, statistically significant differences were observed in low or high PSA density values (*p* = .030), PI-RADS 1–2 and PI-RADS 3-4-5 groups (*p* = .008) and PSA values (*p* = .02), while no statistically significant differences were observed in SUVmax values and uptake patterns (*p* > .05). In multivariate logistic regression analysis, PI-RADS score groups were found to be independent risk factors in predicting malignancy, while age, PSA, PSA density and SUV max were not significant (Table 3).

**Table 1:** General information of patients

Number of Patients	92
Age	66(23–90)
PSA	3.6(0-3198)
Prostate Volume	42(3-216)
PSA Density(ng/mL)	0,07(0,01–56)
PSA Density grup	
PSA density(< 0,15)	49(%86)
PSA density(≥ 0,15)	8(%14)
SUVmax	3,7(1–10)
SUVmax Group	
SUVmax low (< 6)	44(%74,6)
SUVmax high(≥ 6)	15(25,4)
Uptake Pattern	
Focal	65(%70,6)
Diffuse	27(%29,3)
PIRADS score	
PIRADS 1	5(%8,5)
PIRADS 2	52(%88,1)
PIRADS 3	3(%3,4)
PIRADS 4	-
PIRADS 5	-
PIRADS Group	
PIRADS 1–2	65(%70,6)
PIRADS 3-4-5	27(%29,3)
Pathology	
Benign	13(%43,3)
Malign	17(56,7)

PSA: Prostate Specific Antigen, SUVmax: Maximum Standardized Uptake Values, PIRADS: Prostate Imaging Reporting and Data System

**Table 2:** Results of patients who underwent prostate biopsy

	Benign	Malign	CI**	*p*
Number of Patients	13	17		
Age*	65(30–76)	69(46–90)	0,01 − 0,26	0,102
PSA*	5,1(0,08–47)	8,4(3,9-3198)	0–0,10	0,023
Prostate Volume*	51(3-146)	42(16–113)	0,46 − 0,81	0,572
PSA Density(ng/mL)*	0,10(0,01 − 0,35)	0,29(0,06–56)	0–0,10	0,007
PSA Density			0–0,16	0,030
PSA density(< 0,15)	9(%69,2)	5(%29,4)		
PSA density(≥ 0,15)	4(%30,8)	12(%70,6)		
SUVmax*	3,8(1–14)	5,5(1–33)	0,11 − 0,43	0,232
SUVmax Group			0,14 − 0,46	0,283
SUVmax low(< 6)	9(%69,2)	8(%47,1)		
SUVmax high(≥ 6)	4(%30,8)	9(%52,9)		
Uptake Pattern			0,54 − 0,86	0,711
Focal	7(%53,8)	11(%64,7)		
Diffuse	6(%46,2)	6(%35,3)		
PIRADS Score			0–0,10	0,018
PIRADS 1	1(%7,7)	0		
PIRADS 2	8(%61,5)	3(%17,6)		
PIRADS 3	2(%15,4)	1(%5,9)		
PIRADS 4	1(%7,7)	2(%11,8)		
PIRADS 5	1(%7,7)	11(%64,7)		
PIRADS Group			0–0,10	0,008
PIRADS 1–2	9(%69,2)	3(%17,6)		
PIRADS 3-4-5	4(%30,8)	14(%82,4)		

PSA: Prostate Specific Antigen, SUVmax: Maximum Standardized Uptake Values, PIRADS: Prostate Imaging Reporting and Data System, * : Median (Minimum value-Maksimum Value)

**:95% confidence interval (Lower- Upper)

**Table 3:** Multivariant analysis

	Odds Ratio*	*p*
Age	< 0,01	0,998
PSA	0,26 (0,02–2,86)	0,270
PSA density	0,51 (0,06 − 4,49)	0,540
PIRADS	12,06 (1,37–105,93)	0,025
SUVmax	0,84 (0,10 − 7,30)	0,878

Lojistic Regression Analysis

*: 95% confidence interval

## Discussion

Our study showed that only 29.3% of patients with incidentally detected focal increased 18 F-FDG uptake areas in the prostate gland undergoing 18 F-FDG PET-CT examinations for various reasons had histopathological or radiological correlates. Falsely increased 18-FDG uptake was detected in more than two-thirds of the patients. Due to the nature of the central zone of the prostate gland, it may restrict diffusion and can be confused with prostate cancer in mpMRI scans. However, due to its symmetry and location, mpMRI can distinguish it from cancer. In our patient population, incidental 18 F-FDG PET-CT uptake in the central zone of the prostate gland was observed in 4 patients. Although we could not elucidate its pathophysiology, as far as we know, it has been shown in the literature for the first time that 18 F-FDG uptake of the central zone can be incidentally detected using 18 F-FDG PET-CT. In patients who underwent biopsy, low or high SUVmax values and uptake patterns with mpMRI were not statistically significant in detecting prostate cancer. Thus, only the information in the PET-CT report could not determine whether the incidental uptake was malignant or benign. Additionally, low or high PSA density values, PI-RADS scores and PSA values, which are effective in the biopsy decisions of every patient suspected of prostate cancer, were significant in detecting prostate cancer. PI-RADS score groups were found to be independent risk factors in predicting malignancy. PSA density alone is not useful in making decisions in cases where magnetic resonance is controversial [8].

A comprehensive systematic review and meta-analysis including 47,935 patients reported a pooled prevalence of incidental high 18 F-FDG uptake in the prostate of 1.8% (95% CI, 1.3–2.3%) [9]. In another meta-analysis that included 444 patients with incidental uptake, lesions were evaluated in more detail, and 121 of them underwent biopsy. The pooled malignancy risk ranged between 17% and 62%. No significant difference was found in SUVmax between the malignant and benign groups. In a prospective study conducted by Minamimoto et al., TRUS-guided biopsy was performed on areas with focal 18 F-FDG uptake (SUVmax cut-off point > 2.9) and concluded that 18 F-FDG PET-CT has the potential to detect prostate cancer with 80.0% sensitivity [10]. By contrast, Hwang et al. reported incidental recruitment experiences in 120 patients, 23 of whom were diagnosed with prostate cancer via TRUS-guided biopsy after identification of incidental uptake in the prostate; SUVmax was higher in the cancer group (5.7 ± 5.1) than in the benign group (4.8 ± 2.7), but the difference was not statistically significant (*p* = .37) [11]. None of these studies included mpMRI or MRI-targeted biopsy, and they did not include a radiological correlation analysis.

In a study conducted by Anna et al., biopsies were performed on 17 of 32 patients who had incidental 18 F-FDG uptake and mpMRI examination [1]. The standalone detection rate of prostate cancer with 18 F-FDG PET-CT was 0.65. The mpMRI evaluation was performed according to PI-RADS v1, and the total PI-RADS score was not specified in the study. They found that all benign lesions had an SUVmax < 6. This study is the only study other than ours that evaluated mpMRI findings together with 18 F-FDG PET-CT findings. Unlike in our study, they did not use the current PI-RADS v2.1, because PI-RADS v2.1 was published in 2017 and comparisons were made with PI-RADS scores. The comparison of PI-RADS scores using PI-RADS v2.1 is what makes our study significant; PI-RADS v2 has slightly higher sensitivity compared to PI-RADS v1 [12]. Since we used the latest PI-RADS v2.1, we defined lesions in a more practical way and aimed to use a common language with urologists and nuclear medicine physicians.

Although 18 F-FDG PET-CT has low predictive value in the diagnosis of prostate cancer, PSMA PET is the gold standard in the diagnosis of prostate cancer. With the rise of PSMA-based radiotracers, which have shown higher sensitivity and specificity for initial prostate cancer assessment, many studies have highlighted the promising results of combining PSMA PET-CT with mpMRI [13]. This combination is increasingly recommended and may offer a more effective diagnostic approach than using 18 F-FDG PET-CT or mpMRI alone. Pepe et al. showed that 68Ga-PSMA PET-CT compared to mpMRI would prevent 24/30 (80%) planned biopsies, showing a lower false positive rate (20% vs. 43.3%) and a negative predictive value of 85.7% vs. 57.1%, respectively [14]. In another study, Pepe et al. evaluated 125 men with clinical parameters at high risk for PCa with mpMRI and 68Ga-PSMA PET-CT; the accuracy of 68Ga PSMA PET-CT (SUVmax cut-off ≥ 8) in diagnosing csPCa(clinically significant prostate cancer) compared to mpMRI (PI-RADS score ≥ 3) was 92% versus 86.2% [15]. PSMA PET-CT can be recommended in men with suspected PCa who cannot undergo mpMRI.

In our study, only one of the 17 patients with a malignant prostate biopsy had DAC, and the others had acinar adenocarcinoma. Some studies in the literature indicate that 18 F-FDG PET-CT may be a valid tool for diagnosing DAC of the prostate and that it greatly improves the diagnosis of DAC when used in conjunction with PSMA PET-CT, where PSMA PET-CT is inadequate in DAC [16, 17].

The current study has several limitations. First, it was inherently retrospective. Second, 18 F-FDG PET-CT studies were performed to investigate many other primary malignancies but included a limited number of eligible patients with mpMRI and biopsy.

In conclusion, we recommend that men with focal incidental prostate FDG uptake be further evaluated by urologists who can perform a DRE and who typically use biopsy parameters such as serum PSA. Based on the findings in our study, prostate mpMRI can be performed to characterise incidental FDG uptake, thus avoiding unnecessary prostate biopsies.

**Fig. 1:**
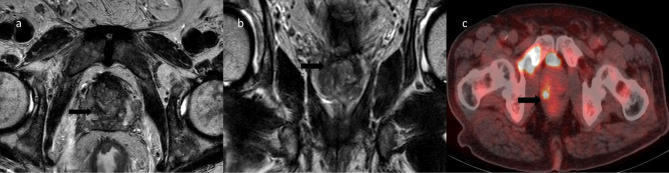
A significant increase in the metabolic activity of the incidental right peripheral prostate focus was detected in a 62-year-old patient who underwent 18F-FDG PET/CT for the staging of metastatic lung cancer and whose serum PSA was 2.36 ng/mL. In prostate mp-MRI, a lesion with an SUVmax of 3.5 was shown in the sagittal T2A MRI (**a**) in the area corresponding to the central zone in the right midapex and in the 18F-FDG PET/CT scan (**c**) in the coronal T2A MRI. It is compatible with the central zone

**Fig. 2:**
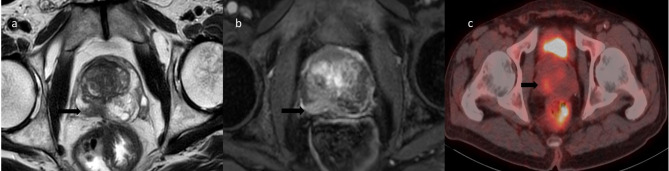
A significant increase in the metabolic activity of the incidental right peripheral prostate focus was detected in a 63-year-old patient who underwent 18F-FDG PET/CT for the staging of rectal cancer and whose serum PSA was 3.92 ng/mL. In prostate mp-MRI, 3 lesions with SUVmax were shown in the sagittal T2A MRI (**a**), in the middle part, in the posterior of the right peripheral zone, in the 18F-FDG PET/CT scan (**c**) in the sagittal post-contrast T1W MRI, and in the mp-MRI PI-RADS 4 lesions are observed. This lesion corresponded to Gleason 3+4 prostate adenocarcinoma on MRI/ultrasound targeted biopsy

## Electronic supplementary material

Below is the link to the electronic supplementary material.

Supplementary Material 1

## Data Availability

No datasets were generated or analysed during the current study.
