# Supplementary material for: Is there a comparable Mp-MRI for incidental prostate uptake on 18 F-FDG PET/CT?
Source: World J Surg Oncol. 2024 Dec 20;22:339. doi: 10.1186/s12957-024-03578-0 (PMC11662805; doi:10.1186/s12957-024-03578-0)
Supplement: Supplementary file 1 — Supplementary Material 1 [file 12957_2024_3578_MOESM1_ESM.pdf]

SCRIBENDI

Certificate of Editing and Proofreading

This certifies that a version of the document titled

Is there a comparable Mp-MRI for incidental prostate uptake on 18F-FDG PET/CT?

authored by

merve şam özdemir

was edited and/or proofread by Scribendi as order number

1018551

for clarity, consistency, and correctness according to the requirements and guidelines specified by the client.

Oct 10, 2024

Scribendi Inc

SCRIBENDI INC.
405 RIVERVIEW DRIVE
CHATHAM, ON N7M 0N3 CANADA
+1 (519) 351 1626

www.scribendi.com

Printed on 2024-10-12 at 11:23 AM
